# Sleep and Reproductive Health

**DOI:** 10.5334/jcr.190

**Published:** 2020-03-23

**Authors:** Olubodun Michael Lateef, Michael Olawale Akintubosun

**Affiliations:** 1 Ladoke Akintola University of Technology, Ogbomoso, NG

**Keywords:** Circadian rhythm, Fertility, Reproductive health, Sex hormones, Sleep deprivation

## Abstract

The reproductive function of humans is regulated by several sex hormones which are secreted in synergy with the circadian timing of the body. Sleep patterns produce generic signatures that physiologically drive the synthesis, secretion, and metabolism of hormones necessary for reproduction. Sleep deprivation among men and women is increasingly reported as one of the causes of infertility. In animal models, sleep disturbances impair the secretion of sexual hormones thereby leading to a decrease in testosterone level, reduced sperm motility and apoptosis of the Leydig cells in male rats. Sleep deprivation generates stressful stimuli intrinsically, due to circadian desynchrony and thereby increases the activation of the Hypothalamus-Pituitary Adrenal (HPA) axis, which, consequently, increases the production of corticosterone. The elevated level of corticosteroids results in a reduction in testosterone production. Sleep deprivation produces a commensurate effect on women by reducing the chances of fertility. Sleeplessness among female shift workers suppresses melatonin production as well as excessive HPA activation which results in early pregnancy loss, failed embryo implantation, anovulation and amenorrhea. Sleep deprivation in women has also be found to be associated with altered gonadotropin and sex steroid secretion which all together lead to female infertility. Poor quality of sleep is observed in middle-aged and older men and this also contributes to reduced testosterone concentrations. The influence of sleep disturbances post-menopausal is associated with irregular synthesis and secretion of female sex steroid hormones.

## Introduction

Sleep deprivation is becoming a common health problem in modern society and many studies are being carried out to investigate its consequences on the growing world. Sleep deprivation can be defined as the partial or almost total absence of sleep that produces various deleterious health challenges in an organism [1]. We live in a sleep-deprived society with several pieces of evidence showing average sleep duration of 6.8 hours daily against 9 hours observed a century ago, and about 30% of adults now sleep for a period less than 6 hours per night [2]. Sleep deprivation is related to common sleep disorders such as insomnia, behaviorally induced insufficient sleep syndrome and obstructive sleep apnea syndrome [3]. A previous report posited that recent sleeping habits of humans notably contradict those of the preindustrial era when humans were able to sleep for longer hours [4]. Sleep deprivation produces adverse effects that human evolution hasn’t been able to build mechanistic adaptations that can compensate for the accumulated sleep debts. The reason behind the persistent sleep deprivation observed among several classes of people is due to pressure to achieve basic socio-economic demands laden by routine work, daily schedules, study, and social engagements. The report of the National Sleep Foundation poll showed that about 39% of respondents reported getting less than seven hours of sleep on weeknights [5]. In addition, research shows that for the past few decades, self-reported sleep durations reduced significantly by approximately 2 hours [5,6]. The normal duration of sleep for an adult is highly controversial, but it is generally implied that about 8 hours of nighttime sleep is sufficient and optimal for general health and wellbeing [7,8,9].

Sleep disorders in both men and women are associated with many health problems like depression, hypertension, glucose deregulation, cardiovascular disease, and anxiety disorders and it was reported that sleep disturbances in women coincide with postpartum depression, pregnancy, menopausal transition and premenstrual dysphoria [10]. The reproductive capacity of animals is affected by alteration of the circadian timing system caused by exposure to irregular light-dark cycles and mutations of main biological clock genes. This results in infertility and abnormalities in menstrual cycle in female shift workers. Infertility is defined as the inability to conceive after 12 months of regular, unprotected intercourse in women less than 35 years of age or after six months in women over 35 years of age. [11]. In animal models, sleep deterioration influenced sexual hormones by lowering the concentration of testosterone and increasing the concentration of progesterone and glucocorticoids in male rats [12] and also, interfered with the male sexual performance [13]. The secretions of all the sex steroid hormones are in synchrony with the circadian rhythm and they regulate sleep patterns [14]. Transition into the menopausal status and the phase changes that occur during the menstrual cycle are as well in synchrony with changes in sleep patterns [15]. Sleep disturbances deregulate the level of steroid hormones in the body and this might lead to infertility among men and women. Cases of infertility are prevalent in the society and several factors have been identified as causes of infertility but little is known about how sleep disturbances modulate the reproductive functions in the body. The focus of this study was to review the effects of sleep deprivation on reproductive hormones and how it affects male and female reproductive health.

## Sleep deprivation

Sleep deprivation (SD) is observed when there is either a total lack of sleep for particular time duration or when there is a shortage of the expected optimal sleep duration. Sleep deprivation is mostly caused by an individual’s contemporary lifestyle and work-related factor such as shift works. Several cases of cardiometabolic and reproductive dysfunctions are reported by shift workers. Disruption of sleep timing patterns by sleeplessness or the fragmentation of sleep [16] may have similar consequences related to severe acute sleep deprivation and this may include; cognitive functions, attention and operant memory [17,18]. Reports from both human and animal models showed that sleep deprivation is connected with many severe physiological consequences such as endocrine disturbances, metabolic, immune and cardiovascular deregulations [19,20,21]. A short duration of sleep in humans is associated with high cases of mortality and a long term sleep deprivation was shown to lead to the death of the animals in an experimental animal model [21,22]. Hence, sleep deprivation is projected as one of the causes of harmful stress that may adversely affect an organism’s well-being and mental health [35].

Sleep deprivation may also be seen as disruption in sleep time during shift work or air travel (jet-lag syndrome resulting from traveling across different time zones) [23]. Sleep deprivation may also set in when people adjust to daylight saving time. Research shows that the longest period of sleep deprivation attained in a human volunteer study was 205 hours (8.5 days). Electroencephalogram (EEG) recording during this sleeping period did not show alpha waves but the EEG signal resembled stage 1 of Non-rapid eye movement (NREM1) sleep during the waking state [24,25].

After a stretched interval of sleep loss, rebound sleep takes place and it is usually longer than the normal sleep duration. Sleep rebound is often accompanied with Rapid eye Movement (REM) sleep and longer periods of the delta-wave, while stage 1 (NREM1) may be absent and stage 2 (NREM2) is shortened [26,27]. The duration of rebound sleep differs markedly from the total duration of sleep loss but it is necessary for sleep to last for several hours more than normal in order to compensate for the accrued sleep debts even within the first 24 hours post-deprivation. The period of sleep deprivation is proportional to the compensatory period and rebound sleep may last for several days. During the compensatory period, the percentage of REM sleep increases above 50%, as a result of increased episodes of REM sleep [28].

## Impaired reproductive function in sleep-deprived male rats

Sleep deprivation is a significant problem among adult men and it has detrimental effects on the male reproductive system in rats [29]. It is generally believed that testosterone secretion changes during the day with highest blood concentration around wake time and lowest during the day [30,31,32]. Several scholarly shreds of evidence prove that SD lowers the concentration of testosterone in the blood and thus, manifesting its deleterious effects on human reproductive health and functions, as well as on the endocrine system [33,34]. Stress is an intrinsic process of sleep restriction that induces many injurious health problems with severe metabolic, endocrinologic and immunologic consequences [35]. Therefore, it is suggested that unknown factors and affected sleep duration associated with sleep deprivation may contribute to differences in sperm motility.

In a study conducted on laboratory rats, after seven days of sleep deprivation, a statistically significant reduction was found in sperm motility when compared to the control group but an insignificant reduction in sperm counts of testis and cauda epididymis was observed between the control and SD group [35]. A significant increase in corticosterone concentrations in the sleep-deprived groups was observed with a corresponding significant decrease in testosterone concentrations in the SD groups. These results are in tandem with several studies that measured steroid hormone levels in the blood of sleep-deprived animals [36,37,38]. High levels of corticosteroid secreted during SD induced by stressful stimuli may suppress the hypothalamus-pituitary-gonadal (HPG) axis resulting in reduced testosterone secretion [39,40]. Circulating testosterone levels increase during sleep [41], and this has been shown to start rising during sleep onset and peak during the first episode of rapid eye movement sleep [42]. Several works of literature on human and animal subjects have shown that SD is associated with reduced levels of androgens in circulation such as testosterone [43] and a decreased level of testosterone can impair gonadal and sexual functions, and ultimately may lead to reduced chances of fertility [44].

**Figure 1:**
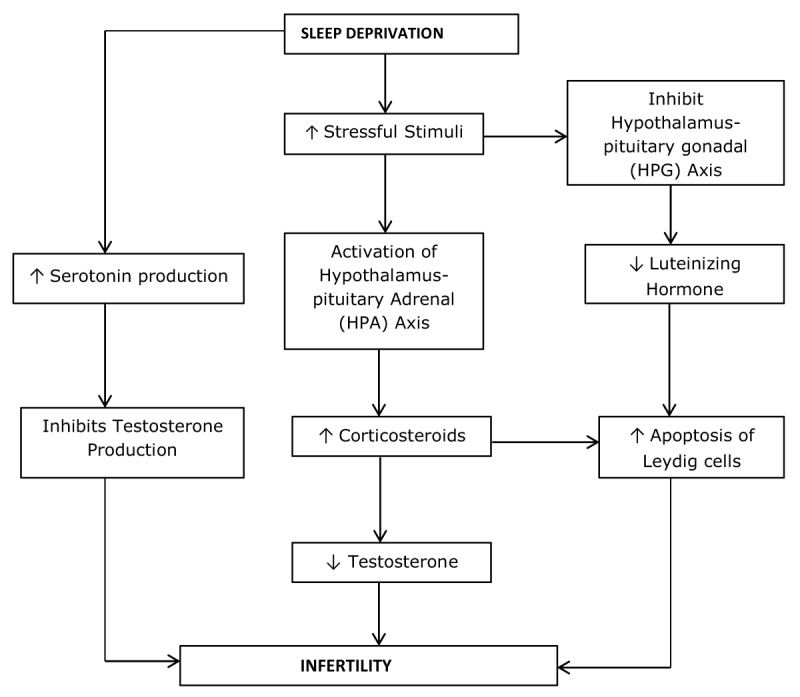
Schematic diagram of the potential mechanism of how sleep deprivation reduces testosterone secretion which eventually leads to male infertility.

The testicular function is regulated by a number of neurotransmitters and neuropeptides, including serotonin (5-HT) [45,46]. Serotonin and its receptors have been localized in Leydig cells isolated from the testes of golden hamsters [47]. Research conducted on animal studies showed that serotonin concentrations are increased during SD [48,49]. Serotonin has been demonstrated to inhibit testosterone production [47]; hence, the reduction in testosterone levels observed during SD may be as a result of serotonin-related inhibition of testosterone production (Figure 1).

More so, decreased production of testosterone may be linked with activation of the hypothalamus-pituitary-adrenal (HPA) axis which increases the concentration of corticosteroid in circulation [50]. SD decreases testosterone level by the inhibition of HPG axis which stimulates the release of corticosteroid. Corticosterone level increases through a negative feedback mechanism that initiates the activation of the HPA axis. Through regulation by the hypothalamus, the pituitary gland produces pituitary gonadotropins such as luteinizing hormone (LH) and follicle-stimulating hormone (FSH). LH stimulates the Leydig cells which have surface receptors for LH, the excited Leydig cells produce and secrete testosterone. However, if corticosterone levels become elevated by stressful stimuli, the release of testosterone in Leydig cells decreases and apoptosis of Leydig cells are induced [51]. In a study by Mazaro and Lamano-Carvalho [52], the neonatal exposure of rats to maternal deprivation and a resultant neonatal stimulation caused a reduction in basal corticosterone levels when compared with a control group but this effect was not observed when the rats were exposed to a new acute stressor of immobilization.

## Effects of sleep deprivation on male sexual behavior

Sleep deprivation also impairs sexual behavior in male rats that previously had excellent sexual behavior. The effect of SD on sexual performance was observed as an increase in latency to initiate intromission behavior and decreased rate of ejaculations and intromissions. This has been reported to constitute lower sexual performance [53]. On the other hand, when the sleep duration was further decreased to mimic the chronic sleep debt present in humans, no significant change in sexual motivation or behavior was observed [54]. Supplementation of testosterone has been observed to be an efficient means of maintaining and improving sexual response in adults and aged male rats [55,56]. There is evidence that the supplementation of testosterone (T) combined with estradiol (E2) shows better results [57]. It is thoughtful to suggest that testosterone replacement administered with or without estradiol or progesterone during the sleep-deprived state could enhance the sexual performances of these animals.

## Age-related impairment to reproductive function in sleep-deprived young men

SD is a known physiological stressor that deregulates the activities of the circadian rhythm. The synthesis of testosterone is dependent on endocrine and neuronal signals which in turn are influenced by physiological conditions such as stress [58]. In a previous study conducted on young men, the concentration of testosterone peaked during sleep either they slept during the day or night [59]. Testosterone concentration was also observed to decrease after waking from both daytime and nighttime sleep. It is worth noting that an equivalent period of sleep rebound after REM-sleep deprivation does not restore testosterone concentration to its baseline levels. This implies that long period of sleep deprivation may result in a long term sexual hormonal imbalances [59]. SD induces stress response by increasing the activity of the hypothalamus-pituitary-adrenal (HPA) axis, which consequently reduces testosterone production [60].

In a study, the sleep of 10 healthy young men was restricted to five hours for eight consecutive days to mimic the condition experienced by at least 15% of the U.S. working population. One week after sleep restriction, a significant decrease of about10 to 15% in daytime testosterone levels was observed when compared with the level found after a normal night of sleep [61]. In a normal sleep episode, another study also observed a lower daytime testosterone levels after one night of total SD as well as after a 4.5-hour sleep episode restricted to the first half of the night. On the contrary, the results from the study also showed that daytime testosterone levels were not affected by two consecutive 4-hour sleep episodes restricted to the second half of the night, which is heavier in REM-sleep [58]. From this standpoint, research suggests that the timing, instead of the adversity of sleep deprivation, plays a crucial role in daytime testosterone concentrations [58].

## Age-related impairment to reproductive function in sleep-deprived aging men

Aging reduces the production of testosterone gradually and this is usually referred to as ‘andropause’. Reduction in testosterone levels is moderate and gradual than that of estrogens during menopause in women [62,63]. Testosterone production progressively reduces beginning from the midlife years at an average rate of 1 to 2% per year [64,65] with relatively reduced sleep duration. Sleep patterns become more interrupted with sleep fragmentations and longer awakenings across the night [66]. NREM sleep when compared to REM sleep is affected by aging because of the reduction in slow-wave sleep (SWS) and a rise in NREM sleep stages during aging [67]. In a study, the relationship between mean overnight blood testosterone concentrations and sleep patterns was investigated in 67 men between the ages of 45 and 74. The findings from the study showed that poorer sleep quality in middle-aged and older men is associated with lower testosterone concentrations. In another clinical trial, 10 young men and 8 older men underwent multisite intensive monitoring, which included simultaneous overnight EEG recordings and frequent blood testosterone sampling (every 2.5 minutes) [68]. A precise correlation between increasing testosterone secretion and deep sleep in young men was observed but no correlation was observed between the sleep stage transitions and testosterone release. The absence of similar results in older men indicates the stronger influence of sleep fragmentation on testosterone levels of older men than young men.

## Sleep Deprivation and changes to Reproductive Hormones in women

Sleep has functional effects on reproductive viability in women of varying ages. During puberty, sleep stimulates the pulsatile secretion of gonadotropin but sleep reduces LH pulse frequency in reproductive age women during the early follicular phase and has negligible consequences in other menstrual cycle phases [69]. Several studies evaluated the impact of sleep on the secretion of sex hormones [70,71,73,101].

## Follicle Stimulating Hormone (FSH)

FSH is an important regulator of reproductive function by stimulating the growth of the ovarian follicle. Sleep plays an important role in the secretion of FSH but there are conflicting results on how sleep affects FSH level. A study conducted on 160 normally cycling, reproductive-age women showed a positive correlation between FSH and sleep duration which continued after adjusting for age and body mass index [72]. However, SD in the second half of the night did not change the level of FSH in women during the early follicular stage of the menstrual cycle [73]. The level of FSH also increases with age and decreases with BMI [74] but this relationship is reversed after menopause because low BMI was shown to be associated with the reduction of nighttime sleep duration after the age of 60 years [75]. A high level of FSH during the early follicular phase of menstrual cycle is a pointer to low ovarian reserve [76] and reproductive aging [77]. The effect of sleep deterioration after menopause is seen as the increased level of FSH with a corresponding reduction in BMI.

## Progesterone

Progesterone regulates the process of the uterine lining and it is essential for implantation and maintenance of pregnancy. Low levels of progesterone may be an index of luteal phase dysfunction [77]. The effects of sleep on progesterone are not well pronounced but it was documented to affect sleep architecture [78]. Low levels of progesterone may be associated with increased sleep-disordered breathing in women with polycystic ovary syndrome (PCOS) [79].

## Thyroid Stimulating Hormone (TSH)

The level of TSH increases during sleep and acute sleep deprivation in healthy young women in their follicular phase is associated with a significant increase in TSH level [80,81]. High level of TSH causes menstrual irregularities, anovulation, amenorrhea and recurrent miscarriages [77]. High level of TSH can stimulate prolactin secretion which may lead to female infertility [82,83,84]. TSH increases before sleep onset and continuously increases throughout the sleep period at night but later decreases during the day [85]. TSH surges during acute SD but becomes diminished during a prolonged SD [85]. The relationship between sleep and TSH shows why acute sleep deprivation may lead to high TSH which consequently, might lead to anovulation, miscarriages, and amenorrhea.

## Luteinizing Hormone (LH)

LH stimulates steroid release from the ovaries, ovulation, and the release of progesterone after ovulation by the corpus luteum [86]. The effects of sleep on menstrual cycle modulate LH secretory pulses and amplitude [85] by diminishing the frequency of LH pulses in the early follicular phase and those awakenings increase the amplitude of LH pulse [87]. The amplitude and frequency of LH pulses further decreases during the mid-follicular phase, where sleep exerts a less noticeable modulation on LH pulses. The amplitude of LH decreases toward the middle to end of the luteal phase without the influence of sleep [73]. The effect of sleep on LH is to down-regulate the activities of LH in both male and female reproductive functions.

## Prolactin (PRL)

Prolactin is a hormone secreted in the pituitary gland and it stimulates milk production (lactation) in women and also plays a role in reproduction. Women with narcolepsy (with or without sleep apnea) have been observed to have lower levels of sleep-related prolactin release [88]. There are inconsistent reports on the effect of sleep on prolactin but sleep disturbances may deregulate prolactin secretion. It was documented that prolactin surges upon sleep onset and is maximal throughout the night [85]. Transient awakening inhibits prolactin secretion and is deeply suppressed by SD [85]. The effect of sleep deprivation results in hyperprolactinemia which is associated with anovulation, polycystic ovary syndrome and endometriosis [89].

## Estradiol

Estradiol is necessary for the development and maintenance of female sex characteristics. It is secreted by the granulosa cells of the ovarian follicles. Estradiol is the primary estrogen during reproductive years and it regulates the activities of FSH and LH and thus, plays an important role in ovulation and growth of the ovarian follicle. The rising and falling of estrogen in the menstrual cycle is necessary for FSH and LH to stimulate ovulation [90]. Estradiol has been reported to increase during partial sleep deprivation in women of reproductive age [73]. The importance of sleep in estrogen secretion was examined in a study and it was observed that women with more variable sleep schedules had higher levels of estradiol than women with more regular schedules [91]. Furthermore, the level of estradiol decreased by 60% in women with regular sleep schedules than women with variable sleep schedules [91]. High levels of estradiol were also observed to be associated with poorer sleep quality [92]. Women with sleep-disordered breathing episodes have been shown to experience better sleep quality with estrogen therapy [93]. The importance of regular sleep schedules can’t be overemphasized in the attainment of a sound and healthy reproductive state in women of different reproductive ages.

## The effects of Melatonin on reproductive health

The endogenous secretion or suppression of melatonin by bright light is implicated in the reproductive health of women. The rate of endogenous secretion of melatonin has similar relationship between the day length and reproductive axis [94]. Melatonin secretion enhances reproductive function by synchronizing sexual behaviors to season and period that are appropriate for mating and conception to take place. High levels of melatonin are found in human ovarian follicular fluid secreted by the pineal gland and ovaries. During ovulation, the importance of melatonin present in the ovarian follicles is to protect the oocyte from oxidative stress [95]. In a study, low levels of follicular melatonin were observed to be associated with higher levels of reactive oxygen species (ROS) and reduced oocyte quality in infertile women [96]. SD reduces the secretion of endogenous melatonin thereby limiting the follicular melatonin levels and thus, exposing the follicles to high influences of oxidative stress. Increasing knowledge of melatonin’s ability to reduce oxidative stress in the ovary has instigated the investigation of exogenous melatonin as a protective agent during in vitro fertilization (IVF) [94]. Melatonin supplementation improves the outcomes of IVF such as the number of oocytes retrieved [97], oocyte quality and maturation [97,98], rate of fertilization [96,98], and the quality of embryo [94,96,98]. Melatonin supplementation has also been shown to improve IVF outcomes in women with PCOS [99]. Sleep deprivation deregulates the endogenous secretion of melatonin and impairs reproductive health.

## Consequences of gestational sleep deprivation on the sexual performance of the offspring

Testosterone is a reproductive hormone that drives sexual behavior and performance in males [100,101,102,103] but little is known about the role of progesterone related with sexual behavior in females. Gestational sleep deprivation affects the sexual behavior of the offspring. In order to ascertain the effects of SD in women similarly to what have been reported in men, a study examined the sexual response of the offspring of female rats who were subjected to sleep restriction during pregnancy and male rats subjected to paradoxical sleep deprivation (PSD) before copulation [54]. It was observed that gestational sleep restriction (SR) compromised sexual behavior in the offspring when they reach adulthood. F1 male offspring of female rats that were subjected to SR throughout the gestational period showed significantly reduced sexual motivation. This was made evident by the increased latency to the first mount and a reduction in the overall number of mounts during the experimental procedure. Similarly, when the male parents were subjected to SR or PSD, the F1 male offspring showed a reduction in testosterone level as well as impaired sexual function. The reduction in sexual motivation observed in the offspring was due to sleep-deprived parents [54]. In humans, both the paternal genetic traits and environmental exposures have profound effects on the offspring’s reproductive health including changes in paternal nutritional, hormonal and metabolic status [104]. The environmental exposures of the father affect the offspring’s phenotype [105] and high levels of ROS decrease sperm mitochondrial respiration [106]. Summarily, the findings from this study show that the sleeping habits of the parents influence the intergenerational transmission of reproduction. Factors such as stress and lack of sleep activate the adrenal-hypothalamus-hypophysis axis, which alters the activity of the aromatase enzyme responsible for the conversion of E2 to T within the hypothalamus [107]. Stress is an inherent part of sleep deprivation and may compromise sexual functions in the offspring and this might be responsible for the alterations in the sexual response observed in the offspring. The detrimental effects of poor sleep quality on the sexual function of the offspring and the health values of quality sleep extend beyond individuals to their descendants.

## Reproductive health of sleep-deprived shift Workers

**Figure 2:**
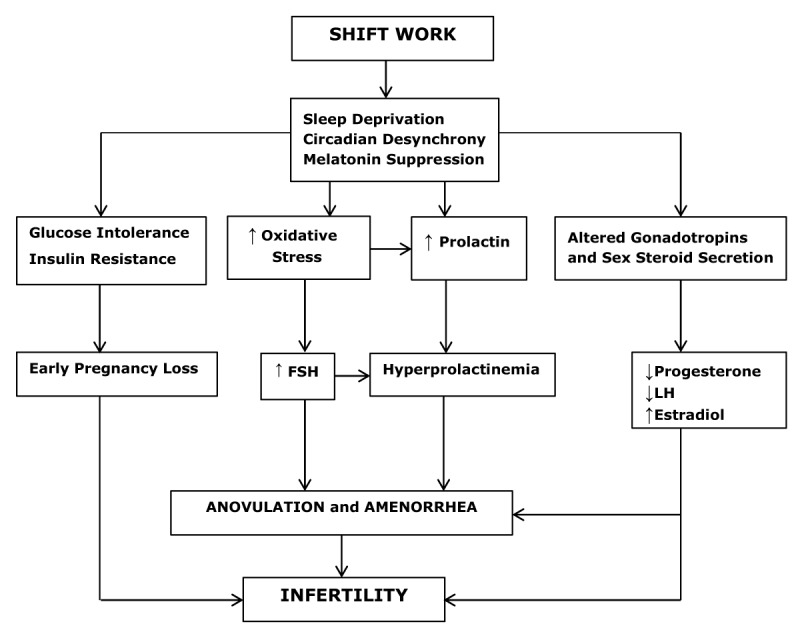
Schematic diagram of how sleep deprivation among female shift workers deregulates the physiological mechanisms of reproduction which substantially contributes to female infertility.

Many studies conducted on the relationship between sleep and circadian rhythm disruption associated with fertility and reproductive health were focused on female shift workers. Shift works are always problematic and involve working at night. The circadian timing system of shift work promotes sleep among the workers and the time allocated for sleep coincides with the time of the high circadian alerting signal. This leads to sleep disruption and misalignment between the externally imposed light-dark cycle and endogenous circadian systems. This alteration in the circadian timing system has negative consequences on physiological function as circadian oscillators are spread throughout the periphery (Figure 2) [108].

A meta-analysis study conducted in 2014 investigated the effect of shift work on reproductive health and came up with the following outcomes. Infertility was defined as time to pregnancy greater than 12 months, early spontaneous pregnancy loss prior to 24 weeks gestation and menstrual cycle disruption defined as cycles less than 25 days or greater than 31 days [109]. An increased odds of menstrual disruption (1.22, 95% confidence interval; 1.15–1.29) and infertility (1.8, 95% confidence interval; 1.01–3.20) was observed among the shift workers but without early spontaneous pregnancy loss. A sub-analysis defined night shift workers as those working shifts only at night for about ten to twelve hours typically beginning between 20:00 and 22:00 [109]. When the sub-analysis was restricted to night shift workers, a significantly increased risk of early spontaneous pregnancy loss was observed which remained after adjustment for confounders (1.41, 95% confidence interval; 1.22–1.63). In a study of 2000 female flight attendants, when the sleep period overlapped with the work times based on the time zone, a significantly increased risk of first-trimester miscarriages was reported among the female flight attendants [110]. Shift work has been used extensively as a driving tool for assessing health risks due to circadian desynchrony but there have been several limitations to these studies. This is because of the differences in shift work schedules and duration of shift work. It is further unclear whether circadian deregulations, sleep deprivation, or exposure to light at night mediate the observed risks. The conflicting results and minimal effect of shift work on early reproductive health may be secondary to the observed health risks and this area needs to be further researched.

## Conclusion

In summary, research reveals that infertility across all ages is affected by the quality, timing, and duration of sleep. Human and animal models clearly show that sleep deprivation alters the level of reproductive hormones that are key players in determining the tendencies of male and female fertility. Findings from this study show that sleeplessness produces physiological alterations similar to oxidative stress which stimulates the activation of the HPA axis and inhibits the HPG axis, thereby resulting in a high level of corticosteroids in the blood. High corticosteroids are implicated in several cases of infertility in men and women. Circadian disruption induced by shift work affects reproductive health by deregulation of sex steroids, gonadotropins and prolactin production. The consequences of a sleep deprived parent can also be passed across to their descendants. The reduction in sexual motivation observed in the offspring may be as a result of sleeplessness experienced during the gestational period. The gatherings of this study show that sleep deprivation not only has detrimental effects on male and female reproduction functions but also transcends to the offspring by impairing their sexual performances. However, mechanisms underlying the complex interaction of sleep on reproductive hormones in women need to be further investigated to enable us understand the clinical approaches to eradicating cases of infertility among female shift workers.
